# Positive Impact of Breastfeeding on Nutritional Status and Metabolic Control in Infants with PKU: A Retrospective Study

**DOI:** 10.3390/nu17172851

**Published:** 2025-09-02

**Authors:** Lizbeth López-Mejía, Sara Guillén-López, Marcela Vela-Amieva, Cynthia Fernández-Lainez, Lilian Castro-Monroy

**Affiliations:** 1 Laboratorio de Errores Innatos del Metabolismo y Tamiz, Instituto Nacional de Pediatría, Secretaría de Salud, Mexico City 04530, Mexico; lizbeth712@hotmail.com (L.L.-M.); mvelaa@pediatria.gob.mx (M.V.-A.); cfernandezl@pediatria.gob.mx (C.F.-L.); 2 Independent Researcher, Mexico City 03630, Mexico; liliancm1305@gmail.com

**Keywords:** inborn errors of metabolism, amino acid metabolism disorders, nutrition, breast milk, infant formula, hyperphenylalaninemia, rare diseases

## Abstract

Background/Objectives: Dietary treatment in phenylketonuria consists of a phenylalanine-restricted diet supplemented with a phenylalanine-free medical formula (Phe-FF). During the first six months of life, phenylalanine requirements can be met with breast milk (BM) or infant formula (IF). Despite all the benefits breastfeeding confers, it is often discontinued upon diagnosis of phenylketonuria, so more evidence is needed to support it. This study aimed to compare the assessments of nutritional status and metabolic control in infants with hyperphenylalaninemia/phenylketonuria who received BM, IF, or a combination of both as sources of intact protein, in addition to Phe-FF. Methods: A retrospective observational study was conducted in hyperphenylalaninemia/phenylketonuria patients between 0 and 6 months of age. Three groups were compared depending on the source of intact protein ingested: (1) BM + Phe-FF; (2) IF + Phe-FF; (3) mixture of BM and IF (BM + IF + Phe-FF). At each clinic visit, an anthropometric assessment and phenylalanine blood levels were analyzed. Results: 185 nutritional and metabolic assessments were included. The lowest median phenylalanine blood concentration was observed in the BM + Phe-FF group (129 µmol/L, interquartile range [IQR]: 39.5–232.5). In the BM + Phe-FF group all assessments were classified as eutrophic: −0.09 (SD ± 0.78); a statistically significant difference was observed between the BMI Z-Score of BM + Phe-FF and BM + IF-Phe-FF (*p* = 0.036). No statistically significant differences were observed in length/age Z-Score. Conclusions: Our results indicate that BM is the best option as a source of intact protein for children under 6 months of age with hyperphenylalaninemia/phenylketonuria, to maintain an adequate nutritional status and metabolic control.

## 1. Introduction

Phenylketonuria (PKU) is one of the most prevalent aminoacidopathies among the inborn errors of intermediary metabolism. Without early diagnosis and treatment, PKU leads to progressive neurodevelopmental impairment, including seizures, self-injurious behavior, autism spectrum disorder, and irreversible intellectual disability [1]. The estimated incidence of PKU in Mexico is 1 in 27,546 screened newborns [2]. At the National Institute of Pediatrics, a national reference center for inborn errors of intermediary metabolism in Mexico, patients with PKU account for approximately 50% of all cases of inborn errors of intermediary metabolism [3].

Timely diagnosis and immediate dietary intervention are crucial in preventing neurological damage. Lifelong dietary management aims to maintain serum phenylalanine (Phe) levels below 360 μmol/L by restricting dietary Phe while ensuring adequate protein intake through a Phe-free medical formula (Phe-FF) [4]. During the first six months of life, phenylalanine (Phe) requirements can be met with breast milk (BM) or infant formula (IF), both of which are supplemented with Phe-free formula (FF) to support optimal growth and development [5]. There are two different ways to provide BM in PKU: one is to quantify it, and the other is to offer it ad libitum after a predetermined amount of Phe-FF.

Breastfeeding is recommended for infants with PKU, as human milk naturally contains lower levels of Phe (46–48 mg/100 mL) and protein (1.07 g/100 mL) [6,7]. In addition to its nutritional profile, BM provides growth factors, hormones, immunological protection, and essential polyunsaturated fatty acids [8]. Numerous studies have demonstrated both short-term and long-term benefits of breastfeeding, including a reduced risk of infections, improved neurodevelopment, and a lower incidence of chronic diseases such as obesity, diabetes, and leukemia [9].

Despite these established benefits, breastfeeding is often discontinued upon diagnosis of PKU [10]. There is a critical need for evidence-based protocols to support breastfeeding in this context, particularly within the Mexican healthcare setting. International guidelines endorse breastfeeding as a viable source of intact protein in PKU management [4], and adequate breastfeeding support by metabolic teams is essential in the early post-diagnosis period [10]. Since the inclusion of PKU in the national newborn screening program in 2011, evaluating treatment practices and outcomes has become increasingly important [2].

Although the nutritional and metabolic outcomes of breastfed infants with PKU have been examined in several countries, the available evidence remains limited. A recent narrative review identified only seven studies that fulfilled the inclusion criteria, with Brazil being the sole representative from Latin America [11]. This scarcity of data underscores a significant geographic and sociodemographic gap in the literature. Additionally, other authors have also highlighted the urgent need for further research on breastfeeding practices in PKU, particularly across diverse populations, to better understand potential regional variations and healthcare system challenges that may affect clinical management and outcomes [11,12].

To our knowledge, no studies conducted in Mexico have systematically compared BM and IF as a source of intact protein in infants diagnosed with PKU. This gap in the literature is particularly relevant given the clinical, nutritional, and public health implications of optimizing feeding practices in this population. Understanding whether BM, IF, or a combination of both, when used in conjunction with Phe-FF, contributes to a more favorable nutritional status and metabolic outcomes is essential for informing evidence-based guidelines and clinical decision-making. Therefore, the objective of the present study was to evaluate and compare the nutritional status and metabolic control of infants with hyperphenylalaninemia (HPA)/PKU who received BM, IF, or a combination of both, in addition to Phe-FF, at a tertiary care center in Mexico. By addressing this question, this study aims to provide novel data from a Latin American context, thereby contributing to the global body of evidence on dietary management in PKU.

## 2. Materials and Methods

### 2.1. Experimental Design and Study Location

A retrospective observational study was conducted on a cohort of Mexican HPA/PKU patients treated at a tertiary care center specialized in inborn errors of intermediary metabolism in Mexico City. Patients aged 0–6 months with a confirmed biochemical and molecular diagnosis of HPA/PKU due to phenylalanine hydroxylase deficiency were classified into three biochemical phenotype groups based on their previously determined genotype [13,14] and the highest pre-treatment blood Phe level: classical PKU (cPKU; Phe 1200 μmol/L), mild PKU (mPKU; Phe 600–1200 μmol/L), and mild HPA (MHP; Phe 120–600 μmol/L) [15]. The decision to restrict Phe from the diet and initiate Phe-FF treatment was made when Phe levels were equal to or above 360 μmol/L. Patients receiving Phe-FF as part of their nutritional management were included. As part of their treatment, all patients were instructed to provide a measured amount of Phe-FF, explicitly calculated for each infant, followed by breastfeeds directly from the breast ad libitum. The Phe-FF amount was calculated to meet approximately 80% or less of the 2–3 g/kg/day protein requirement for patients aged 0–6 months, which aligns with the total protein intake recommended in the PKU nutritional management guidelines of the Southeast Regional Genetics Network-HRSA Region 3 (SERN) and the GMDI [16]. Titration of only Phe-FF was performed based on blood Phe levels to maintain serum Phe at 360 μmol/L or less. During each clinic visit, a 3-day dietary recall was conducted to document total Phe-FF intake in grams and milliliters (total and per feeding), as well as the timing and number of breastmilk feedings. None of the infants included received complementary foods during this period. Patients with tetrahydrobiopterin (BH4) disorders or those undergoing BH4 (sapropterin) therapy were excluded. According to our institutional care protocols for HPA/PKU, an informative session and a brochure are provided to parents or caregivers explaining the benefits of breastfeeding at diagnosis.

Patients were categorized into three groups depending on the source of intact protein complementary to the Phe-FF they were ingesting during their first evaluation post-treatment until 6 months of age: Group (1) breast milk and Phe-free formula (BM + Phe-FF), (2) infant formula and Phe-free formula (IF + Phe-FF), (3) mixture of breastmilk and IF (BM + IF + Phe-FF). In this last group, patients used IF or BM alternately during these six months, along with Phe-FF.

### 2.2. Procedure

At each scheduled clinic visit, every patient underwent a standardized evaluation that included anthropometric measurements, as well as determination of blood Phe concentrations. Data from these evaluations were systematically retrieved from the medical records of patients spanning the period from 2009 to 2025, ensuring longitudinal follow-up throughout early growth and metabolic management. For this study, baseline assessments obtained at the time of diagnosis were excluded from the analysis to focus on outcomes observed during treatment and follow-up.

### 2.3. Anthropometric Evaluation

The anthropometric evaluation included weight and length for each subject. It was executed by two standardized metabolic dietitians who weighed and measured the patients in duplicate (without clothing) using a Seca 354^®^ pediatric digital scale (Hammer Steindamm 3-2522089, Hamburg, Germany) and a Seca 416^®^ infantometer (Hammer Steindamm 3-2522089, Hamburg, Germany) using the World Health Organization (WHO) techniques [17]. Values were reported to the nearest 0.1 kg and 0.1 cm.

The body mass index (BMI) was calculated from the anthropometric data. The BMI Z-score (BAZ) and length-for-age Z-score (LAZ) indicators were obtained using Anthro^®^ software (Version 3.2.2.1, Geneva, Switzerland). According to the WHO classification for children aged 0–5 years, a BAZ score below −2 SD was considered as underweight, a score between −2 and +2 SD was considered eutrophic, a score above +2 SD was considered overweight, and a score above +3 SD was considered obese. A LAZ greater than or equal to −2 SD was considered normal.

### 2.4. Blood Phenylalanine Determination

To determine blood Phe concentration, blood drops were deposited and dried on filter paper, and then processed using the previously described tandem mass spectrometry (MS/MS) technique [18]. All samples were obtained two hours after the patients were fed.

### 2.5. Statistical Analysis

The statistical analyses were performed with GraphPad Prism software (version 10.4.2, San Diego, CA, USA). The Shapiro–Wilk test was used to assess the distribution of the data. When data had a parametric distribution, one-way ANOVA, mixed-effects analysis with Geiser–Greenhouse correction, and Dunnett’s multiple comparison tests were conducted; the results were presented as the means ± SDs. The Mann–Whitney U test was used for nonparametric data, followed by Dunn’s multiple comparisons adjustment test, with results presented as medians (Q1–Q3). A *p*-value < 0.05 was considered statistically significant (* *p* < 0.05, ** *p* < 0.01, *** *p* < 0.001, **** *p* < 0.0001).

### 2.6. Ethical Considerations

This study was conducted in accordance with the guidelines of the Declaration of Helsinki. It was approved by the Institutional Review Board (Ethics, Research, and Biosafety Committees) of the National Institute of Pediatrics (protocol codes 2021/056 and 2020/014).

## 3. Results

A total of 31 patients with HPA/PKU participated in the study. Their genotype, HPA/PKU classification, and distribution based on the source of intact protein consumed are summarized in Table 1. The BM + Phe-FF group included 13 patients (41.9%), the IF + Phe-FF group 11 patients (35.5%), and the BM + IF + Phe-FF group seven patients (22.6%).

The Phe maximum historical blood concentrations before treatment were 1433 μmol/L for the BM + Phe-FF group, 1504 μmol/L for the IF + Phe-FF group, and 1235 μmol/L for the BM + IF + Phe-FF group. The highest proportion of patients with cPKU was in the IF + Phe-FF group (10/11, 91%), followed by the BM + Phe-FF group (8/13, 61.5%) and the BM + IF + Phe-FF group (3/7, 42.8%).

A total of 185 nutritional and metabolic assessments were conducted in infants, comprising 20 females (64.5%) and 11 males (35.5%). Assessments were conducted on average for four months (1–6 months) after diagnosis. Each patient was followed longitudinally, with a minimum of two and a maximum of fifteen evaluations per patient. The assessments were distributed across the three feeding groups as follows: 77/185 (41.6%) from the BM + Phe-FF 89% group, 48/185 (25.9%) from the IF + Phe-FF group, and 60/185 (32.4%) from the BM + IF + Phe-FF group.

Before diagnosis, 26 out of 31 (83.8%) infants were breastfed. After diagnosis, breastfeeding continued in 20/31 (64.5%), indicating a substantial reduction of breastfeeding post-diagnosis.

### 3.1. Metabolic Control

**Figure 1:**
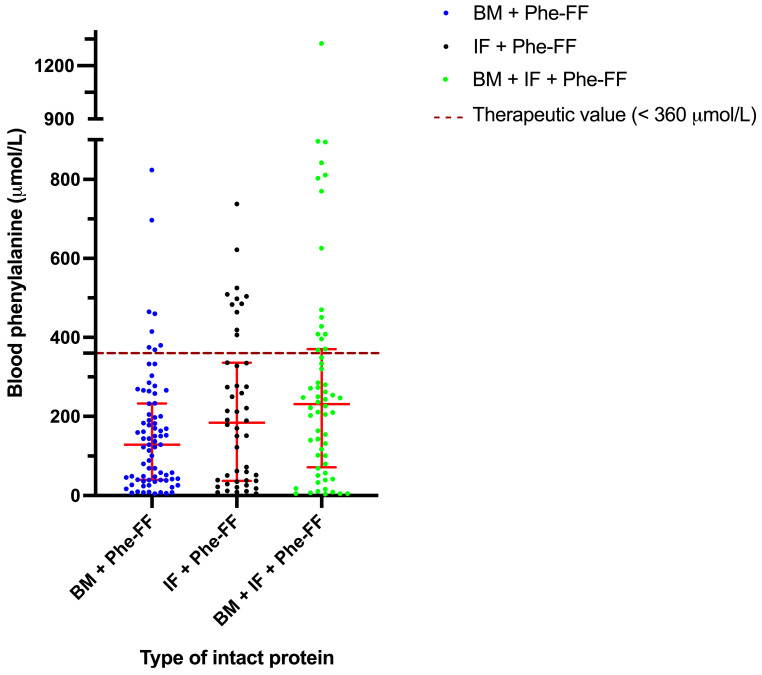
Blood phenylalanine (Phe) concentrations in HPA/PKU patients in the studied groups: Breast milk with Phe-free formula (BM + Phe-FF), Infant formula with Phe-free formula (IF+ Phe FF), and mixture group with Breast milk + Infant formula + Phe-free formula (BM+ IF+ Phe FF). Data are presented as median ± interquartile range.

Phe blood concentrations across the three feeding groups are shown in Figure 1. All groups maintained median Phe levels below the recommended threshold of 360 µmol/L, indicating overall adequate metabolic control. However, the lowest median Phe concentration was observed in the BM + Phe-FF group (129 µmol/L, interquartile range [IQR]: 39.5–232.5), followed by the IF + Phe-FF group (184 µmol/L, IQR: 37.3–335.8), and the highest in the BM + IF-Phe-FF group (231 µmol/L, IQR: 71.7–370.3). A statistically significant difference was found between the BM + Phe-FF and BM + IF-Phe-FF groups (*p* = 0.0018), with the BM group exhibiting tighter control of Phe. The proportion of assessments with Phe levels in the target range (<360 µmol/L) also differed by group: 89% in the BM + Phe-FF group, 77.1% in the IF + Phe-FF group, and 73.3% in the BM + IF-Phe-FF group (Figure 1).

### 3.2. Anthropometric Outcomes

#### 3.2.1. Length-for-Age Z-Score

**Figure 2:**
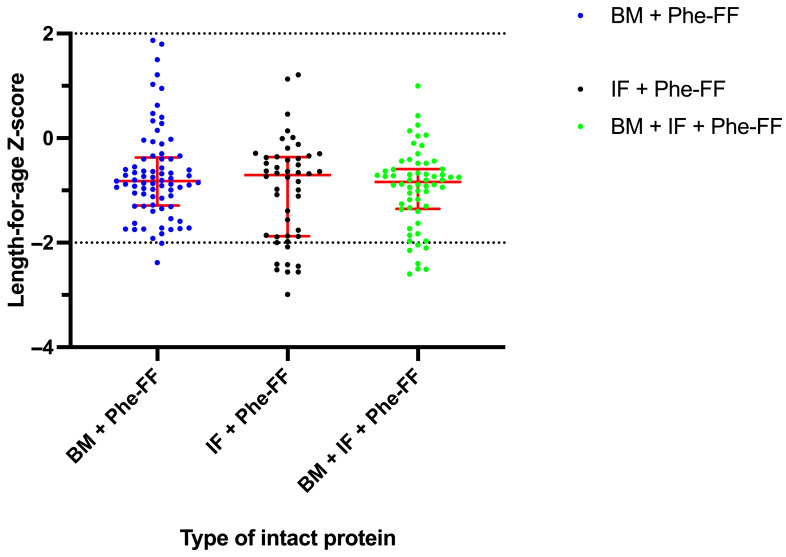
Length-for-age Z-score (LAZ) in HPA/PKU patients in the studied groups: Breast milk with Phe-free formula (BM+ Phe-FF), Infant formula with Phe-free formula (IF+ Phe FF), and mixture group with Breast milk + Infant formula + Phe-free formula (BM+ IF+ Phe FF). Data are presented as median ± interquartile range. Normal range is depicted in dash lines.

No statistically significant differences were observed in LAZ between the feeding groups (Figure 2). Mean LAZ scores were: BM + Phe-FF group −0.69 (standard deviation [SD] ± 0.87), IF + Phe-FF group −0.99 (SD ± 0.98), and BM + IF-Phe-FF group −0.96 (SD ± 0.75). Across all assessments, 17/185 (9.2%) showed LAZ scores < −2 SD, indicating stunting. The proportion of assessments within normal LAZ range (>−2 SD) was: 75/77 (97.4%) for the BM + Phe-FF group, 40/48 (83.3%) for the IF + Phe-FF group, and 40/48 (83.3%) for the BM + IF-Phe-FF group.

#### 3.2.2. BMI-for-Age Z-Score

**Figure 3:**
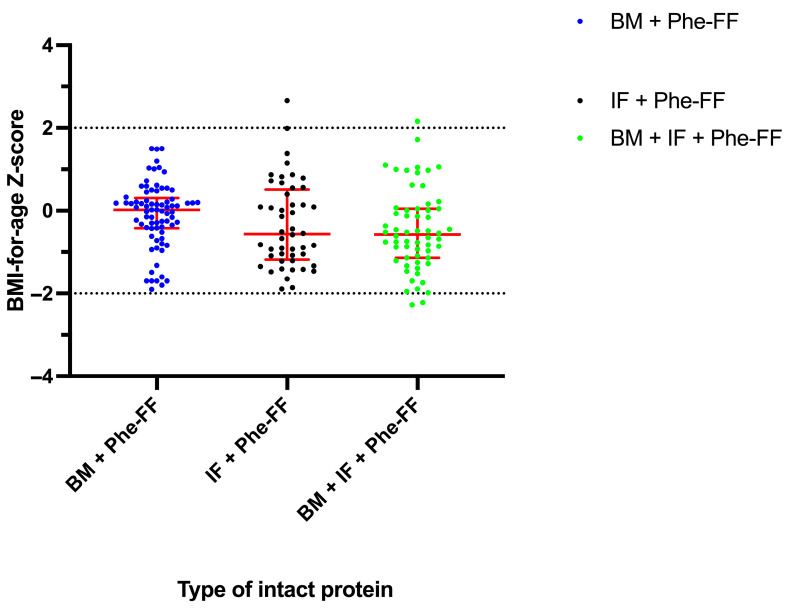
BMI-for-age Z-score (BAZ) in HPA/PKU patients, by group: Breast milk with Phe-free formula (BM+ Phe-FF), Infant formula with Phe-free formula (IF+ Phe FF), and mixture group with Breast milk + Infant formula + Phe-free formula (BM+ IF+ Phe FF). Data are presented as median ± interquartile range. Normal range is depicted in dash lines.

Figure 3 shows the BAZ across groups. Mean BAZ was: BM + Phe-FF group: −0.09 (SD ± 0.78), IF + Phe-FF group: −0.33 (SD ± 1.04), BM + IF-Phe-FF group: −0.47 (SD ± 0.97). A statistically significant difference was observed between the BM + Phe-FF and BM + IF-Phe-FF groups (*p* = 0.036), with the BM + Phe-FF group showing a trend toward more favorable BMI status. In terms of nutritional classification: In the BM + Phe-FF group, all assessments (77/77 (100%) were classified as eutrophic. The IF + Phe-FF group had 1/48 (2.1%) assessment classified as overweight, and 47/48 (97.9%) were eutrophic. Within the BM + IF-Phe-FF group, 1/60 individual (1.7%) was classified as overweight, 2/60 (3.3%) as underweight, and the remaining 57/60 (95%) as eutrophic.

## 4. Discussion

To the best of our knowledge, this is the first study conducted in Mexican patients with HPA/PKU that compares BM + Phe-FF versus IF + Phe-FF and a combination of BM + IF-Phe-FF as sources of intact protein. One of the key findings of this study was that patients who received BM as the primary source of intact protein had the lowest median blood Phe levels compared to those in the BM + IF-Phe-FF group and the BM + IF-Phe-FF group. These results suggest that BM, when combined with Phe-FF, may contribute to improved metabolic control and treatment adherence. This observation is consistent with recent international guidelines, which recommend and support breastfeeding in babies with phenylalanine hydroxylase deficiency [4]. It is also noteworthy that most biochemical phenotypes in the total cohort correspond to cPKU (67.7%), a finding also observed in the BM + Phe-FF group (61.5%). Taken together, these findings reinforce the feasibility and potential benefits of breastfeeding, even in infants with the most severe PKU phenotypes.

These findings are consistent with those reported in other studies. For example, Kose et al. reported that serum Phe levels were significantly lower in breastfed infants with PKU compared to non-breastfed infants with PKU [19]. Also, another study of 173 patients with severe and moderately severe PKU exhibited lower Phe levels than those who were never breastfed [20]. In a recent narrative review of breastfeeding in patients with PKU, seven observational studies were included. They found no differences in mean serum Phe concentrations between the BM and IF groups during follow-up [11]. Additionally, the breastfeeding group had the lowest percentage of Phe assessments above the therapeutic target of 360 μmol/L, which is consistent with the findings reported by Banta-Wright et al. [21], who, in a retrospective study, observed that more breast-fed infants with PKU had Phe concentrations within the normal range.

Ilgaz et al. performed a meta-analysis of fifteen studies that included 507 BM + Phe FF fed PKU infants, observing adequate growth [12], similar to the results in our research. Although no statistically significant differences were observed in LAZ, it is noteworthy that the breastfeeding group exhibited a higher proportion of assessments reflecting normal linear growth. This trend is consistent with previous studies, which have demonstrated that breastfed infants tend to achieve superior growth outcomes, particularly in terms of height, compared to their counterparts who receive infant formula [22].

The detection of a small proportion of overweight cases in the IF + Phe-FF and BM + IF-Phe-FF groups, compared to the breastfed group, where all individuals were classified as eutrophic, may suggest a potential protective effect of breastfeeding against early excessive weight gain. These findings align with previous reports indicating that PKU infants fed with IF or BM + IF-Phe-FF tend to exhibit faster weight gain and higher BMI trajectories. Furthermore, the protective role of breastfeeding in reducing the risk of overweight and obesity later in life has been well documented in the literature [23,24].

Additionally, it is known that regardless of the method of BM administration, blood Phe control and growth are not significantly different [25]. The advantage of administering BM directly from the mother’s breast and after ingestion of Phe-FF is that it allows for the regulation of satiety signals in the infant, which may have contributed to normal BMI assessments. Moreover, this type of administration is associated with a longer duration of breastfeeding [25]. These findings suggest that breastfed PKU infants achieved adequate growth and nutritional status, comparable to or better than the other groups in our study.

However, various situations call for the use of IF, such as insufficient breast milk production, feeling that the infant is not consuming enough calories, fear of high Phe levels, or the mother being unable to continue breastfeeding because she is a working mother. Banta-Wright et al. have described similar challenges faced by mothers of patients with PKU in maintaining breastfeeding [26]. Pinto et al. discussed a common situation where the blood Phe concentration is high at diagnosis, often requiring a temporary suspension of breastfeeding for 24 to 48 h. Restarting breastfeeding is very challenging for mothers, who are often overwhelmed and distressed, and frightened that giving more BM could potentially harm their babies, especially in severe cases like ours [27]. Situations like this could lead to a decrease in breastfeeding rates after diagnosis.

The percentage of breastfed children after diagnosis in this study was 64.4%, which is lower than the 81% previously reported from Canada and the USA [10], but closer to the 61% reported by other European centers [19]. Breastfeeding maintenance is more complicated in PKU [27]. Possibly, the decrease in breastfed children after diagnosis in our study was related to the severity of the disease, as the majority of our cohort had a classical form, and it has been previously described that maternal stress could contribute significantly [10].

This study has some limitations, such as a small sample size and those inherent to the retrospective study design. Likely, a selection bias occurred to ensure a well-defined, representative population of cPKU and mPKU patients. We included MHP because, years ago, PKU treatment was solely based on a Phe-restricted diet. However, new therapies have since emerged and become available to patients at our institution. Prospective studies would probably focus more on cPKU and mPKU. A multicenter prospective study should be conducted to increase sample sizes and allow for more comprehensive data collection. Additionally, medical records have transitioned from physical to electronic formats, so some information might be incomplete. We were unable to measure feelings, stress, and mothers’ perceptions regarding BM in PKU, but this is something we plan to explore in future studies.

A comprehensive clinical history gathering all information about breastfeeding and mothers’ perceived barriers, especially in cPKU, should be mandatory in every clinic managing PKU infants to better understand and support breastfeeding. Clear and concise information, along with guidance at different stages, should be provided to all families with PKU patients, along with education for the healthcare team to promote breastfeeding, boost their confidence, and encourage its continuation for as long as possible. Additionally, involving a Lactation Consultant could be beneficial [28].

Further research is needed to clarify the determinants of breastfeeding continuation and duration beyond 6 months in patients with HPA/PKU, as well as the long-term effects of breastfeeding on this population. This knowledge will guide effective educational and policy interventions that support and encourage prolonged human milk feeding in this vulnerable group.

## 5. Conclusions

In this study, no significant differences were found between groups of BM + Phe-FF, IF + Phe-FF, and BM + IF-Phe-FF of fed infants regarding nutritional status and metabolic control. Median Phe levels in all groups were below 360 mmol/L, with the lowest median identified in the breastfeeding group and a higher percentage of Phe blood concentrations within the target range. We also observed a higher rate of normal assessments in terms of LAZ and BAZ in the breastfed group compared to the IF + Phe-FF and BM + IF-Phe-FF. According to our findings, we consider BM to be the best option as a source of intact protein for children under 6 months of age with HPA/PKU, in terms of maintaining an adequate nutritional status and metabolic control of Phe levels.

The findings of this study have relevant clinical implications for the dietary management of infants with HPA/PKU. Although no statistically significant difference was observed between the feeding groups, infants who received BM in combination with Phe-FF demonstrated the most favorable outcomes, including lower median blood Phe concentrations, a higher proportion of values within the therapeutic target range, and more frequent normal assessments of linear and ponderal growth. These results suggest that BM, when complemented with Phe-FF, represents not only a safe but also an optimal source of intact protein for infants younger than six months of age with HPA/PKU.

From a clinical perspective, promoting and supporting breastfeeding in this population may enhance metabolic control, improve adherence to dietary treatment, and contribute to better overall nutritional status, even in cases of cPKU. These findings reinforce current international guidelines recommending breastfeeding for infants with phenylalanine hydroxylase deficiency and provide evidence from a Latin American context, where data have traditionally been limited. Incorporating these results into clinical practice highlights the importance of counseling caregivers, training healthcare providers, and ensuring the availability of Phe-FF to support breastfeeding as a key part of dietary management in PKU during early infancy.

## Figures and Tables

**Table 1:** Genotype, HPA classification and study group of patients with HPA/PKU.

Patient	Blood Phe Max Historical (μmol/L)	Genotype c.DNA	Protein	Phenotype	Group
1	448	c.[434A>T];[809G>A]	p.[Asp145Val];[Arg270Lys]	MHP	BM + Phe-FF (N = 13)
2	890	c.[1162G>A];[1162G>A]	p.[Val388Met];[Val388Met]	mPKU	
3	1154	c.[1066-11G>A];[1162G>A]	p.[?];[Val388Met]	mPKU	
4	964	c.[165del];[208_210del]	p.[Phe55Leufs*6];[Ser70del]	mPKU	
5	1008	c.[809G>A];[1162G>A]	p.[Arg270Lys];[Val388Met]	mPKU	
6	1280	c.[1A>T];[1066-11G>A]	p.[Met1?];[?]	cPKU	
7	1900	c.[1A>T];[1066-11G>A]	p.[Met1?];[?]	cPKU	
8	1900	c.[60+5G>T];[441+5G>T ]	p.[?];[?]	cPKU	
9	1454	c.[809G>A];[809G>A]	p.[Arg270Lys];[Arg270Lys]	cPKU	
10	2176	c.[60+5G>T];[1315+1G>A]	p.[?];[?]	cPKU	
11	2506	c.[1045T>C];[1045T>C]	p.[Ser349Pro];[Ser349Pro]	cPKU	
12	1696	c.[682G>A];[682G>A]	p.[Glu228Lys];[Glu228Lys]	cPKU	
13	1252	c.[649T>C];[649T>C]	p.[Cys217Arg];[Cys217Arg]	cPKU	
14	1559	c.[60+5G>T];[441+5G>T]	p.[?];[?]	mPKU	IF + Phe-FF (N = 11)
15	1066	c.[208_210del];[728G>A]	p.[Ser70del];[Arg243Gln]	cPKU	
16	1900	c.[441+5G>T];[1055del]	p.[?];[Gly352Valfs*48]	cPKU	
17	1217	c.[625_626insC];[625_626insC]	p.[Ile209Thrfs*6];[Ile209Thrfs*6]	cPKU	
18	1423	c.[168+5G>C];[1066-11G>A]	p.[?];[?]	cPKU	
19	1527	c.[1A>T];[1042C>G]	p.[?];[Leu348Val]	cPKU	
20	1527	c.[1A>T];[1042C>G]	p.[?];[Leu348Val]	cPKU	
21	1351	c.[1042C>G;1238G>A];[1042C>G;1238G>A]	p.[Leu348Val;Arg413His];[Leu348Val;Arg413His]	cPKU	
22	1715	c.[60+5G>T];[1315+1G>A]	p.[?];[?]	cPKU	
23	1634	c.[60+5G>T];[1162G>A]	p.[?];[Val388Met]	cPKU	
24	1623	c.[722G>A];[842+1G>A]	p.[Arg241His];[?]	cPKU	
25	467	c.[60+5G>T];[1169A>G]	p.[?];[Glu390Gly]	MHP	BM + IF + Phe-FF (N = 7)
26	401	c.[721C>T];[1066-11G>A]	p.[Arg241Cys];[?]	MHP	
27	909	c.[60+5G>T];[1241A>G]	p.[?];[Tyr414Cys]	mPKU	
28	966	c.[814G>T];[1282C>T]	p.[Gly272*];[Gln428*]	mPKU	
29	1753	c.[791A>G];[1315+5_1315+6insGTGTAACAG]	p.[His264Arg];[?]	cPKU	
30	2825	c.[116_118del];[1045T>C]	p.[Phe39del];[Ser349Pro]	cPKU	
31	1325	c.[60+5G>T];[1066-11G>A]	p.[?];[?]	cPKU	

Abbreviations: MHP, mild hyperphenylalaninemia; mPKU, mild PKU; cPKU, classical PKU; BM + Phe-FF, breast milk and Phe-free formula group; IF + Phe-FF, infant formula and Phe-free formula; BM + IF + Phe-FF, mixture of breastmilk and infant formula. Symbols: * stop codon; ? unknown effect.

## Data Availability

The original contributions presented in the study are included in the article; further inquiries can be directed to the corresponding author.

## Abbreviations

The following abbreviations are used in this manuscript:

BMI.	body mass index
HPA.	hyperphenylalaninemia
PKU.	phenylketonuria
Phe.	phenylalanine
WHO.	World Health Organization
SERN.	Southeast Regional Genetics Network
GMDI.	Genetic Metabolic Dietitians International
BH4.	tetrahydrobiopterin
LAZ.	length for age Z-Score
BAZ.	BMI for age Z-Score
BM.	breastmilk
IF.	infant formula
Phe-FF.	phenylalanine free formula
IQR.	interquartile range
cPKU.	Classic Phenylketonuria
mPKU.	Mild Phenylketonuria
MHA.	Mild hyperphenylalaninemia
